# Straw-returning of Triticale to field improved the yield of foxtail millet efficiently by modulating soil physicochemical traits and fungal microbiome assembly

**DOI:** 10.3389/fmicb.2026.1791055

**Published:** 2026-03-11

**Authors:** Guohong Yu, Ya Han, Jingqiang Liu, Yingying Zhang, Hongbo Hao, Mingzhe Li

**Affiliations:** 1 Key Laboratory of Crop Drought Tolerance Research of Hebei Province, Institute of Dry Farming, Hebei Academy of Agriculture and Forestry Sciences, Hengshui, China; 2 Hebei Center for Ecological and Environmental Geology Research, Hebei GEO University, Shijiazhuang, China; 3 State Key Laboratory of Wheat Improvement, College of Life Sciences, Shandong Agricultural University, Tai'an, China

**Keywords:** foxtail millet, fungal community, rotation model, soil properties, straw-returning of Triticale

## Abstract

Green manure-crop rotation systems are effective management practices for maintaining soil health and enhancing crop yield. However, the influence of various green manure-millet rotation systems on soil properties, fungal community structure, and millet yield in the North China Plain remains undetermined. In this study, three types of green manures with foxtail millet rotation experiment were conducted. The physico-lchemica indexes, millet yield and soil fungal community characteristic were detected. Our findings suggest that three green manure-millet rotation systems increased millet yield compared to millet-winter fallow (Si-Le). Among them, the Triticale-millet rotation (Si-Ts) showed the highest yield increase, with a rise of 46.16% in 2021 and 85.7% in 2022. In 2021, compared with Si-Le, the organic matter (OM) in Si-Ts increased by 17.86%, and the available phosphorus (AP) rose by 113.82%. In 2022, in contrast to Si-Le, the alkali-hydrolyzed nitrogen (AHN) in Si-Ts increased by 17.68%, the available phosphorus (AP) by 37.56%, and the available potassium (AK) by 12.56%. Additionally, Si-Ts exhibited the highest diversity of soil fungi and the greatest relative abundance of beneficial genera from the dominant phylum Ascomycota and Mortierellomycota. Moreover, Green manure rotations (particularly Si–Ts) alleviate these constraints by simultaneously augmenting microbial diversity (driven by OM/AK/AHN) and crop yield (driven by AP/OM). Overall, the Triticale-millet rotation is a feasible practice for improving soil conditions, maintaining soil microbial balance, and ensuring high yields of millet. Our findings offer theoretical support for green manure-crop rotation in influencing the soil environment and the sustainable development of the millet industry in the North China Plain.

## 1 Introduction

China is a largely agricultural country, and 30 % of its grain production comes from only 7% of the world’s cultivated land (Hou et al., 2021). In northern China, the arid climate is becoming increasingly severe, which seriously affects agricultural production (Wang and Zhang, 2024). In China, the low-plain region of Hebei is currently undergoing severe groundwater over-extraction, and it has become one of the largest areas of groundwater depletion in the world (Guan et al., 2023). In order to scientifically establish a mechanism for sustainable farmland protection, in 2016, the Ministry of Agriculture and nine other ministries jointly issued the “Pilot Program for Exploring the Implementation of a Crop Rotation and Fallow System”. This program designated North China as a pilot area for seasonal fallow. It changed the traditional two-season annual cropping system to a single-season cropping system. The aim was to reduce the area of winter wheat that heavily relies on groundwater irrigation. Instead, rainfed crops such as millet and corn are grown during the warm season, thereby reducing groundwater usage (Ren, 2019; Zhang et al., 2023). Thus, crop rotation and fallow system can be considered as the most practical measure to maintain soil fertility and increase crop yield in this region.

Foxtail Millet (*Setaria italica*) is a traditional staple crop in China, having a long history of cultivation. It is drought-resistant, tolerant of poor soils, and resilient to acidic and alkaline conditions, making it an important component of sustainable dryland ecological agriculture in both the arid northern and southern regions of China (Zhao et al., 2024). The planting of green manure can not only improve soil fertility but also concurrently boost the supply of nutrients for crops (Tejada et al., 2008; Pittelkow et al., 2015). Incorporation of green manure, combined with surface mulching of crop residues can alleviate damage to soil structure, reduce surface runoff and soil losses, and preserve soil moisture (Singh et al., 2016; Singh et al., 2017). Furthermore, green manure-crop rotations can boost soil nutrients cycling (e.g., C, N, P), enhance their availability, and increase main crop yields (Ding et al., 2021; Sun Q. et al., 2024; Sun Y. F. et al., 2024; Damon et al., 2014). For instance, crop rotations can restrain soil erosion by increasing land utilization rate and land cover, promote endogenous nitrogen fixation, provide nutrients from green manure, and augment soil microbial activities and diversity, thereby the yield and quality of subsequent crops can be improved (Hao et al., 2024; Bai et al., 2022; Kim et al., 2012). Green manure-maize rotation, as opposed to continuous maize treatment, can significantly increase maize yield and also cause changes in soil nutrients in the surface layer of the soil (Yang et al., 2023). Xu et al. It have reported that in comparison with fallow treatment, the application of green manure exerts a positive influence on soil pH, nitrogen availability, soil microbial growth and activity (Xu H. et al., 2023; Xu J. et al., 2023). Hence, it is suggested that the green manure-millet rotation system should be employed in the low plain area of Hebei province, where groundwater is over-extraction. However, the research regarding the impacts of diverse varieties of green manure on both millet yield and soil properties throughout the growth and incorporation phases of green manure remains scarce.

Soil microorganisms play a crucial part in the cycling of carbon and nitrogen within the soil and they are of great significance in maintaining soil health and facilitating sustainable agricultural development (Li B. et al., 2023; Li W. H. et al., 2023; Zhao et al., 2016). Soil fungi are an indispensable part of the soil microorganism community and act as prominent decomposers in the soil ecological system. They are closely linked to nutrient uptake, organic matter decomposition, and disease mechanisms, ultimately influencing plant yield (Mei et al., 2022). Crop diversity, exemplified by the practice of crop rotation, exerts diverse influences on the structure of soil fungal communities. Numerous research investigations have demonstrated that, in contrast to a continuous cropping system, a rotation regime is capable of furnishing a more favorable environment conducive to promoting a higher diversity and greater abundance of fungi (Wang et al., 2022; Khan et al., 2019; Li et al., 2014). Through the alternation of crop species, the composition and abundance of fungal taxa can be significantly modulated, potentially enhancing soil health and fertility (Sun Q. et al., 2024; Sun Y. F. et al., 2024). The response of soil fungal communities to different green manure-millet rotations and the relationship between soil fungi and soil properties on the North China Plain remains elusive. While specific studies directly addressing green manure-millet rotations in the North China Plain are limited, related research provides insights into broader trends and potential mechanisms.

In this study, we thoroughly examined how the rotation of millet with various green manures, specifically Triticale, rapeseed, and February orchid, impacted millet yield, soil properties, and the fungal communities in the surface soil. The primary aim was to precisely define the effects of green manure-millet rotations on these aspects and to enhance our understanding of the complex relationship between fungal communities and soil properties. We hypothesized that green manure-millet rotation systems would play d a regulatory role in determining millet yield, soil properties, and fungal communities. The findings of this study have significantly improved our comprehension of the benefits that green manure-millet rotations bring to soil fungal communities. These insights offer a profound understanding of the connection between different green manure types and soil conditions, ultimately influencing the productivity of major crops. Moreover, we were able to select the optimal crop rotation patterns between green manure and millet in the North China Plain.

## 2 Materials and methods

### 2.1 Experimental site, soil sampling and millet yield

The green manure–millet rotation experiment was initiated in 2017 in Hujiachi Town, Hengshui City, Hebei Province (37°44′N, 115°47′E). The site lies within a warm temperate continental monsoon zone, with a mean annual temperature of 12.4 °C and approximately 550 mm of annual precipitation, most of which occurs from June to September. The area experiences roughly 200 frost-free days per year, and the accumulated temperature above 0 °C reaches 4,863 °C. The soil is classified as loam fluvo-aquic. In the 0–20 cm layer, basic soil characteristics include 2.0 g·kg^−1^ organic matter, 70.66 mg·kg^−1^ alkali-hydrolyzable nitrogen, 33.81 mg·kg^−1^ available phosphorus, and 190.52 mg·kg^−1^ available potassium.

Four rotation schemes were established, each arranged in triplicate: (1) Millet (*Setaria italica*) monocropping (millet–fallow, Si-Le)—summer millet grown following a winter fallow period; (2) Triticale–millet rotation (millet–Triticale, Si-Ts)—summer millet alternated with Triticale; (3) Rapeseed–millet rotation (millet–rapeseed, Si-Bn)—summer millet combined with winter rapeseed; (4) February orchid–millet rotation (millet–February orchid, Si-Ov)—summer millet paired with winter February orchid.

The summer millet variety Henggu 13 was sown annually in late June at 7.5 kg·hm^−2^ in row-seeding patterns. Harvesting took place on September 25, after which all straw was returned to the field. Triticale (Zhongsi 1,048) was seeded in early October at sowing rate of 150 kg·hm^−2^ and incorporated into the soil through rotary tillage the following May. Rapeseed (Hengyou 8) and February orchid (conventional variety) were broadcast between millet rows at the end of August each year at rates of 15 kg·hm^−2^ and 45 kg·hm^−2^, respectively, and were likewise incorporated through rotary tillage in mid-May. All treatments followed uniform field management practices except for the identity of the winter crop.

From each plot, five soil cores were systematically collected and combined to form a composite sample. Samples were transported to the laboratory in insulated containers with ice packs to ensure preservation. Upon arrival, each composite soil sample was passed through a 2-mm sieve to homogenize the soil matrix. The sieved soil was then divided into two distinct portions: one portion was stored at −80 °C for subsequent DNA extraction and molecular analysis while the other portion was air-dried under controlled conditions (Soil samples should be kept under conditions where the temperature is controlled at 35 °C ± 5 °C for 4 ~ 5 days.) to facilitate comprehensive physiochemical property analysis. Upon the maturation of millet, grain yields were quantified by conducting a single harvest per plot. Subsequent laboratory analyses measured yield components, encompassing ear diameter, panicle weight, grain weight, grain weight per spike, and thousand-seed weight.

### 2.2 Soil property analysis

Soil pH was determined by mixing with distilled water at a soilto-solution ratio (1:2.5 g·mL^−1^) (Mclean, 1982) and electrical conductivity (EC) was measured using electrode method (Miller and Curtin, 2006). Soil organic matter (OM) was used by potassium dichromate oxidation with external heating (K_2_Cr_2_O_7_-H_2_SO_4_) (Metherell, 1992). The concentrations of soil nutrients were assessed using specific analytical techniques. The alkaline hydrolyzable nitrogen (AHN) content was measured via the alkaline hydrolysis diffusion method (Sha et al., 2014). For available phosphorus (AP) determination, the sodium bicarbonate extraction coupled with molybdenum antimony resistance spectrophotometry was employed (Page et al., 1982). As for available potassium (AK), it was extracted using ammonium acetate and subsequently quantified through flame photometry (Halajnia et al., 2009).

### 2.3 DNA extraction and sequencing

DNA was isolated from soil samples using a commercially available extraction kit (DP336, Tiangen Biotech Co., Ltd., Beijing). The integrity and quality of the extracted DNA were verified through 1% agarose gel electrophoresis. Custom-designed primers incorporating barcodes were synthesized to target the specific region of interest for sequencing. These primers were then purified using the Agencourt AMPure XP nucleic acid purification system to ensure high-quality templates for subsequent sequencing analyses. In the process of soil DNA extraction, single-stranded DNA fragments were initially created through NaOH-induced denaturation. Following this, the Miseq sequencing platform, supplied by Orivison Biotechnology Co., Ltd., Beijing, was utilized to generate the DNA sequences of the aforementioned template fragments. After obtaining the raw sequencing data, a stringent quality control protocol was applied to filter and refine the sequences, thereby yielding high-quality, optimized sequences. These optimized sequences were subsequently subjected to clustering analysis, resulting in the formation of Operational Taxonomic Units (OTUs). Each OTU was meticulously annotated to elucidate the taxonomic information of the microbial communities within the soil samples, thus providing valuable insights into the soil’s microbial diversity and composition (Rognes et al., 2016). Based on the clustering results, Alpha diversity analysis (Amato et al., 2013; Miller et al., 2016) and Beta diversity analysis were performed (Jiang et al., 2013). Taxonomic information at various levels was obtained from the annotation results, allowing for analyses of community composition and differences between samples (Segata et al., 2011). Sequencing data are available in the Sequence Read Archive (SRA) database at NCBI (accession number: PRJNA733687).

### 2.4 Data processing

The soil properties—pH, electrical conductivity (EC), organic matter (OM), Alkali-hydrolyzale nitrogen (AHN), available phosphorus (AP), and available potassium (AK)—as well as millet yield and yield components, were subjected to one-way analysis of variance (ANOVA) using SPSS 21. All differences and correlation coefficients were regarded as statistically significant at *p* < 0.05. Furthermore, non-metric multidimensional scaling (NMDS) was employed to evaluate the diversity of fungal communities under different millet rotation treatments. The relationships between environmental factors and fungal community diversity across crop rotation systems were examined using redundancy analysis (RDA) and Significance of constrained axes was tested by 999 Monte-Carlo permutations. Pearson correlation analysis and Mantel tests were performed with the ChiPlot online tool.^1^

## 3 Results

### 3.1 Soil properties and millet yield

Soil chemical properties and millet yield under different crop rotation regimes in 2021 and 2022 were summarized in Table 1. In 2021, both the Si-Ts (millet–Triticale) and Si-Bn (millet–rapeseed) rotations exhibited significantly higher soil organic matter (OM) and available phosphorus (AP) contents than the Si-Le (millet–winter fallow) system. Specifically, OM increased by 17.86 and 16.33% in the Si-Ts and Si-Bn systems, respectively, while AP rose markedly by 113.82% in Si-Ts and 46.16% in Si-Bn. In 2022, the Si-Ts rotation showed pronounced enhancements in alkaline hydrolyzable nitrogen (AHN), available phosphorus (AP), and available potassium (AK) relative to Si-Le, with increases of 17.68, 37.56, and 12.56%, respectively. Compared with the millet–winter fallow system, green manure–millet rotations substantially improved millet grain yield (Table 1) and associated yield components (Supplementary Table S1).

**Table 1:** Soil chemical properties and millet yield under different crop rotation systems.

Year	Rotation systems	pH	EC (μs·cm^−1^)	OM (%)	AHN (mg·kg^−1^)	AP (mg·kg^−1^)	AK (mg·kg^−1^)	Millet yield (kg·hm^−2^)
2021	Si-Ts	7.98 ± 0.16a	145.97 ± 3.30ab	2.31 ± 0.06a	94.97 ± 0.76a	30.94 ± 1.31a	179.77 ± 2.46b	5676.45 ± 179.716a
	Si-Bn	8.10 ± 0.20a	150.40 ± 2.08a	2.28 ± 0.03a	85.29 ± 1.81b	21.15 ± 1.14b	143.59 ± 0.23c	4801.01 ± 358.05b
	Si-Ov	8.03 ± 0.20a	142.27 ± 2.08b	2.02 ± 0.07b	86.26 ± 2.06b	16.31 ± 0.92c	139.68 ± 0.0d	4609.94 ± 21.69bc
	Si-Le	7.92 ± 0.33a	146.97 ± 5.25ab	1.96 ± 0.01b	93.76 ± 0.76a	14.47 ± 1.09c	206.14 ± 1.59a	4151.38 ± 171.99c
2022	Si-Ts	8.30 ± 0.25a	128.87 ± 14.39a	2.08 ± 0.32a	89.44 ± 5.50a	18.57 ± 3.68a	135.30 ± 0.07a	5152.34 ± 70.84a
	Si-Bn	8.34 ± 0.07a	113.73 ± 6.86a	1.78 ± 0.24a	74.94 ± 9.24b	14.08 ± 1.01b	120.42 ± 3.6b	3739.77 ± 337.60b
	Si-Ov	8.26 ± 0.04a	114.40 ± 8.82a	1.79 ± 0.28a	75.74 ± 4.97b	14.21 ± 1.91b	121.60 ± 3.4b	4150.21 ± 268.21b
	Si-Le	8.32 ± 0.10a	120 ± 9.08a	1.8 ± 0.05a	76 ± 4.94b	13.5 ± 1.50b	120.2 ± 4.37b	2774.96 ± 419.75c

Different lowercase letters indicate significantly differences at the *P* < 0.05. Si-Ts, millet– Triticale; Si-Bn, millet–rapeseed; Si-Ov, millet–February orchid; Si-Le, millet–fallow. EC, electrical conductivity; OM, Soil organic matter; AHN, alkaline hydrolyzable nitrogen; AP, available phosphorus; AK, available potassium.

In 2021, the yield followed the order Si-Ts > Si-Bn > Si-Ov (millet–oat) > Si-Le. The Si-Ts treatment achieved the highest yield (5676.5 kg·hm^−2^), corresponding to a 36.7% increase over Si-Le, whereas the Si-Bn system enhanced yield by 15.6%. Moreover, relative to Si-Le, the Si-Ts rotation significantly increased ear diameter and thousand-grain weight by 5.00 and 10.60%, respectively. The grain output rate under Si-Bn was 3.91% higher than that of Si-Le. The Si-Ov rotation significantly improved panicle weight, grain weight per spike, and valley output rate by 13.72, 20.35, and 5.82%, respectively. In 2022, a similar yield trend was observed (Si-Ts > Si-Ov > Si-Bn > Si-Le), with Si-Ts again producing the highest yield (5152.3 kg·hm^−2^), representing an 85.7% increase over Si-Le. Yield gains of 34.8 and 49.6% were also recorded in the Si-Bn and Si-Ov systems, respectively. Compared with Si-Le, the Si-Ts rotation significantly enhanced panicle weight, grain weight per spike, and thousand-grain weight by 17.06, 39.41, and 14.34%, respectively. Additionally, the Si-Ov system significantly increased panicle weight and grain weight per spike by 35.88 and 20.46%, respectively.

### 3.2 Soil fungal community

**Figure 1:**
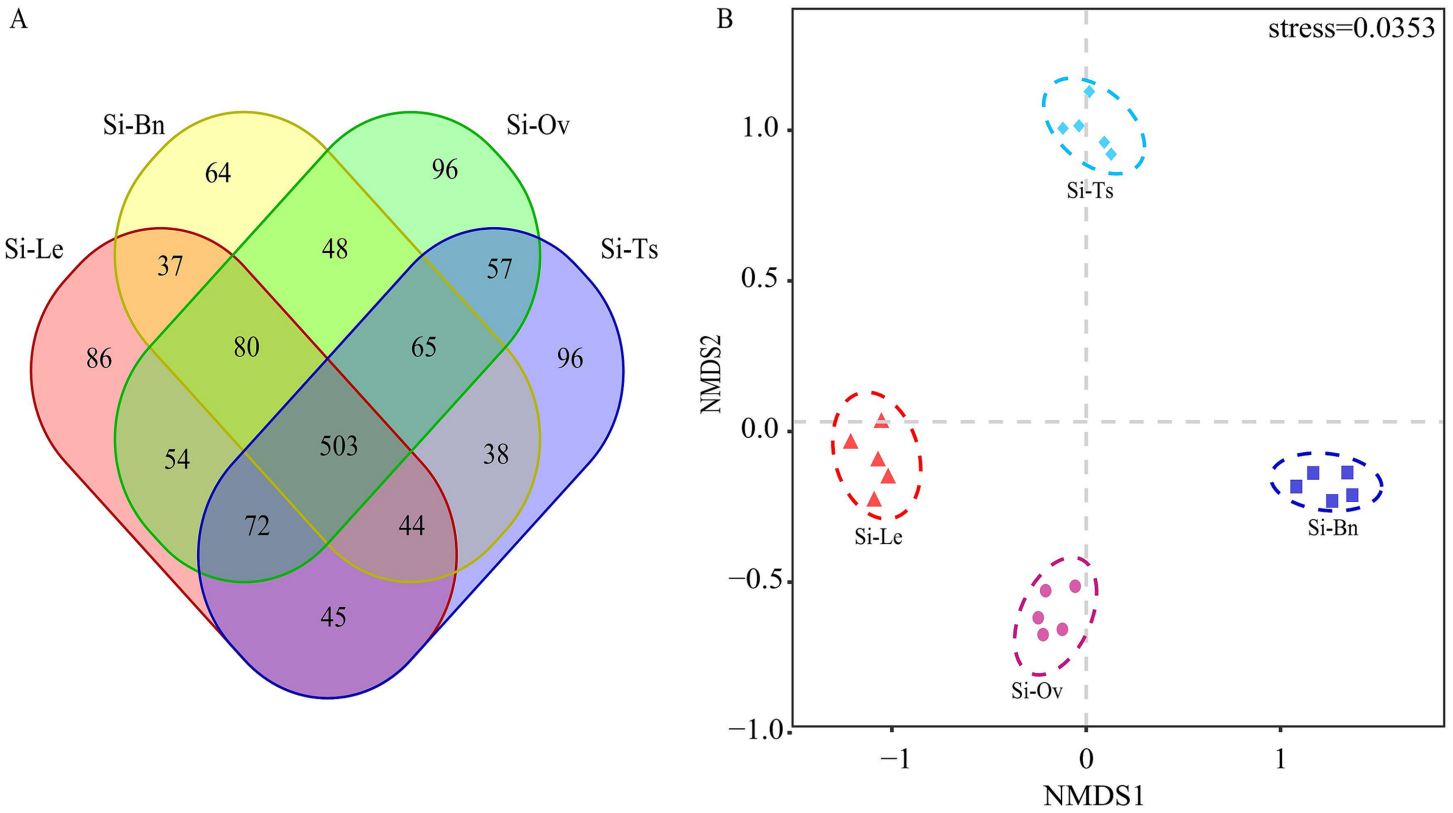
Differences in soil fungal OTUs across crop-rotation systems. Venn diagram: colors distinguish rotations; numbers within each oval give the total OTUs for that rotation; overlaps indicate OTUs shared among rotations **(A)**. NMDS of *α*-diversity: axes represent relative distances only and lack intrinsic biological meaning **(B)**.

Differences in fungal operational taxonomic unit (OTU) distributions among the various crop rotation regimes were visualized using a Venn diagram (Figure 1A). OTU composition differed markedly among the four rotation systems, with green manure treatments harboring distinct microbial assemblages compared with the millet-winter fallow control (Si-Le). Specifically, the comparisons revealed 513, 212, and 472 unique OTUs in the Si-Ts, Si-Ov, and Si-Bn systems, respectively, when contrasted with Si-Le. To further evaluate variations in fungal community structure among treatments, beta diversity analysis was conducted (Figure 1B). Each ellipse encompassed five replicate samples corresponding to the same rotation system, and the tight clustering of samples within ellipses indicated low intra-group variability. In contrast, the absence of overlap between ellipses from different treatments reflects clear compositional differentiation among rotation systems. Overall, the 20 samples were effectively grouped according to crop rotation type, demonstrating strong experimental reproducibility and greater inter-group than intra-group variation, thereby confirming the suitability of the dataset for subsequent analyses.

**Figure 2:**
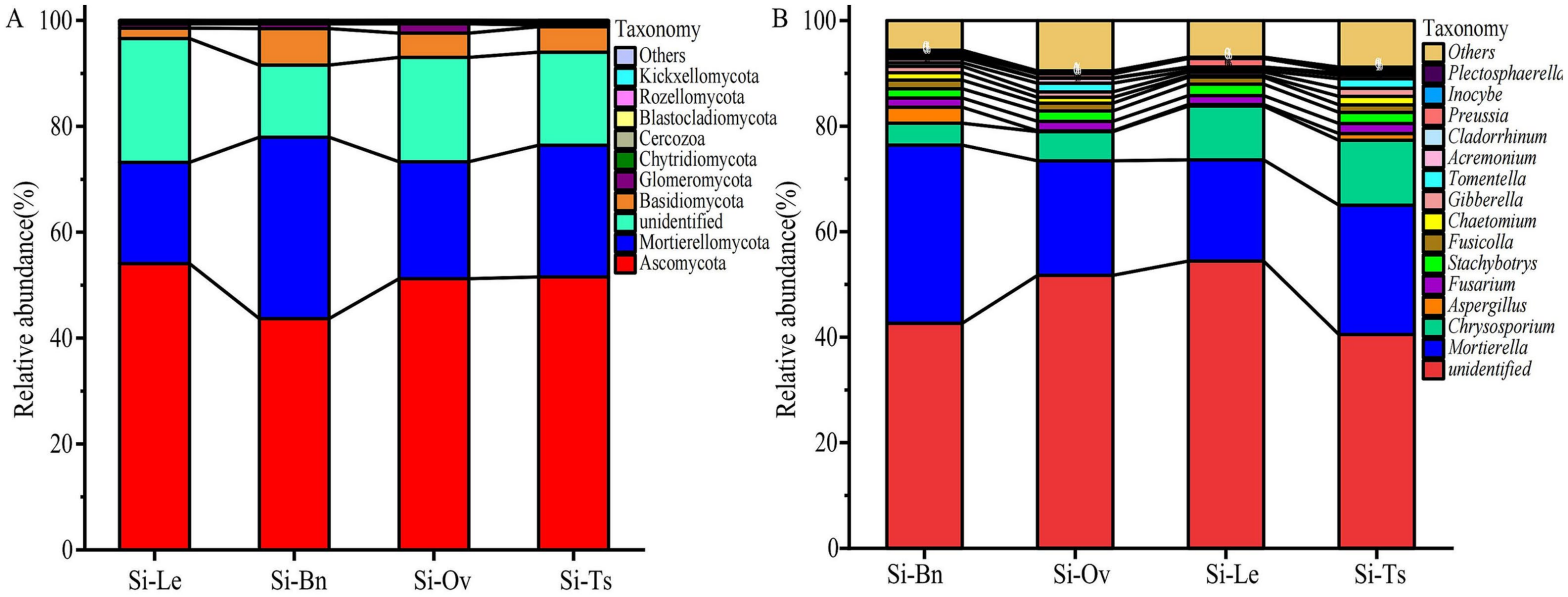
Relative abundance of fungal community composition at the phylum **(A)** and genus **(B)** levels. Si-Le, millet–fallow; Si-Bn, millet–rapeseed; Si-Ov, millet–February orchid; Si-Ts, millet–triticale.

For all crop rotation treatments, the fungal community was predominantly composed of the phyla Ascomycota and Mortierellomycota, which together represented approximately 70.6–79.0% of the total relative abundance. Among these, Ascomycota consistently exhibited the highest dominance, accounting for 42.6–55.4% of the fungal assemblages in the four rotation systems, whereas Mortierellomycota ranked second with relative abundances ranging from 17.1 to 35.2%. Ascomycota, with its relative abundance averaging 54.1% under Si-Le, significantly higher than the 43.7% recorded for Si-Bn. A similar pattern was observed for Mortierellomycota, whose abundance in Si-Bn (34.3%) exceeded that in Si-Le (19.1%) by 15.2 percentage points (Figure 2A). Compared to Si-Le, *Mortierella* was higher 13.1, 27.8 and 75.9% in Si-Ov, Si-Ts and Si-Bn, respectively. *Chrysosporium* was higher 20.4% in Si-Ts (Figure 2B).

By comparing the relative abundance of fungal genera across different planting patterns, the results showed that, compared to the Si-Le group, the relative proportion of *Mortierella* increased by 33.7, 85.1, and 19.2% in the Si-Ts, Si-Bn, and Si-Ov groups, respectively. The relative proportion of *Gibberella* in the Si-Ts, Si-Bn, and Si-Ov groups was significantly higher by 94.4, 50.0, and 39.0% compared to the Si-Le group. The relative proportion of *Inocybe* in the Si-Ts and Si-Bn groups was significantly higher than that in the Si-Le group by 12.1 and 14.2%, respectively. In the Si-Ts group, the relative proportions of *Nigrospora*, *Neopaxillus*, *Bionectria*, *Wallemia*, and *Clavulicium* were significantly higher than those in the Si-Le group by 1.5 times, 9.6 times, 4.2 times, 13.4 times, and 6.8 times, respectively. In contrast, the relative proportion of *Microdochium* in the Si-Ts, Si-Bn, and Si-Ov groups was significantly reduced by 49.1, 45.1, and 27.4% compared to the Si-Le group (Supplementary Table S2).

### 3.3 Correlation analysis

**Figure 3:**
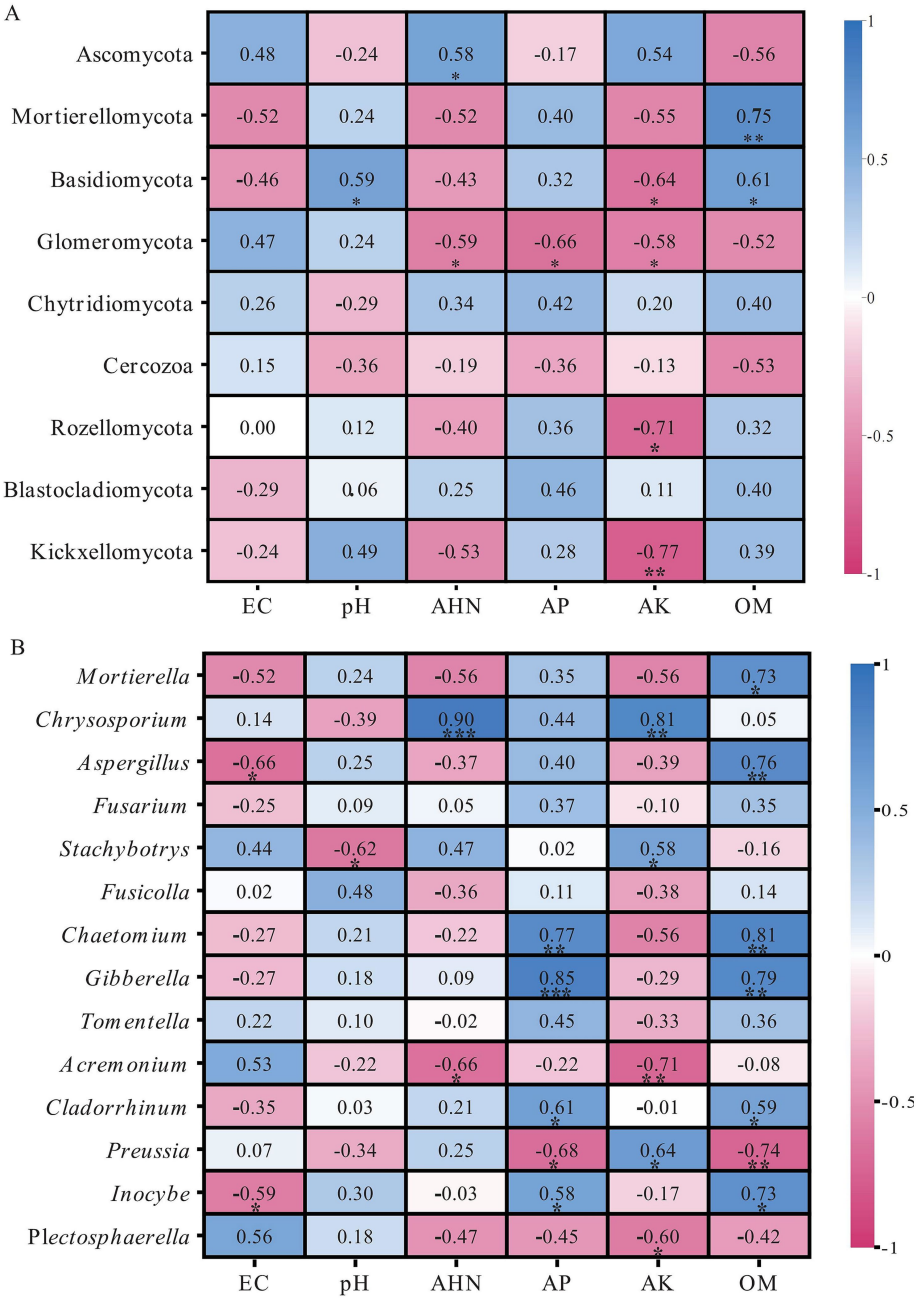
Pearson correlations between soil properties and the diversity of fungal communities at the phylum **(A)** and genus **(B)** levels. EC, electrical conductivity; OM, soil organic matter; AHN, alkaline hydrolyzable nitrogen; AP, available phosphorus; AK, available potassium (* *p* < 0.05, ** *p* < 0.01, *** *p* < 0.001).

Pearson correlation analysis was conducted to examine the relationships between soil chemical properties and fungal community diversity at the phylum level under different crop rotation systems. Soil organic matter (OM) showed strong positive correlations with Mortierellomycota (*r* = 0.76, *p* < 0.01) and Basidiomycota (*r* = 0.61, *p* < 0.05), while exhibiting significant negative relationships with unidentified fungi (*r* = −0.83, *p* < 0.001). Available potassium (AK) was negatively correlated with several dominant phyla, including Glomeromycota (*r* = −0.58, *p* < 0.05), Rozellomycota (*r* = −0.71, *p* < 0.05), and Kickxellomycota (*r* = −0.77, *p* < 0.01), and positively correlated with unidentified fungi (*r* = 0.68, *p* < 0.05). Alkaline hydrolyzable nitrogen (AHN) showed a significant positively correlated with Ascomycota (*r* = 0.58, *p* < 0.05) and negative correlation with Glomeromycota (*r* = −0.59, *p* < 0.05). Additionally, Soil pH was positively associated with Basidiomycota (*r* = 0.59, *p* < 0.05), whereas available phosphorus (AP) showed a significant negative correlation with Glomeromycota (*r* = −0.66, *p* < 0.05). Overall, these results indicate that soil nutrient availability and organic matter content are key drivers shaping fungal community composition (Figure 3A). In the genus level, Soil organic matter (OM) was positively correlated with *Mortierella* (*r* = 0.75, *p* < 0.05), *Aspergillus Chaetomium* (*r* = 0.76, *p* < 0.01)*, Gibberella* (*r* = 0.81, *p* < 0.01) *and Inocybe* (*r* = 0.79, *p* < 0.01), and was significantly negatively correlated with *Preussia* (*r* = 0.74, *p* < 0.01). Available phosphorus was significant positively correlated with *Chaetomium* (*r* = 0.77, *p* < 0.01) and *Gibberella* (*r* = 0.85, *p* < 0.01), and was significantly negatively correlated with *Preussia* (*r* = −0.68, *p* < 0.05). Available potassium was positively correlated with *Chrysosporsium* (*r* = 0.81, *p* < 0.01) and *Preussia* (*r* = 0.64, *p* < 0.05). Alkali-hydrolyzed nitrogen was significantly positively correlated with *Chrysosporsium* (*r* = 0.90, *p* < 0.01), and was significantly negatively correlated with *Acremonium* (*r* = −0.66, *p* < 0.05) (Figure 3B).

**Figure 4:**
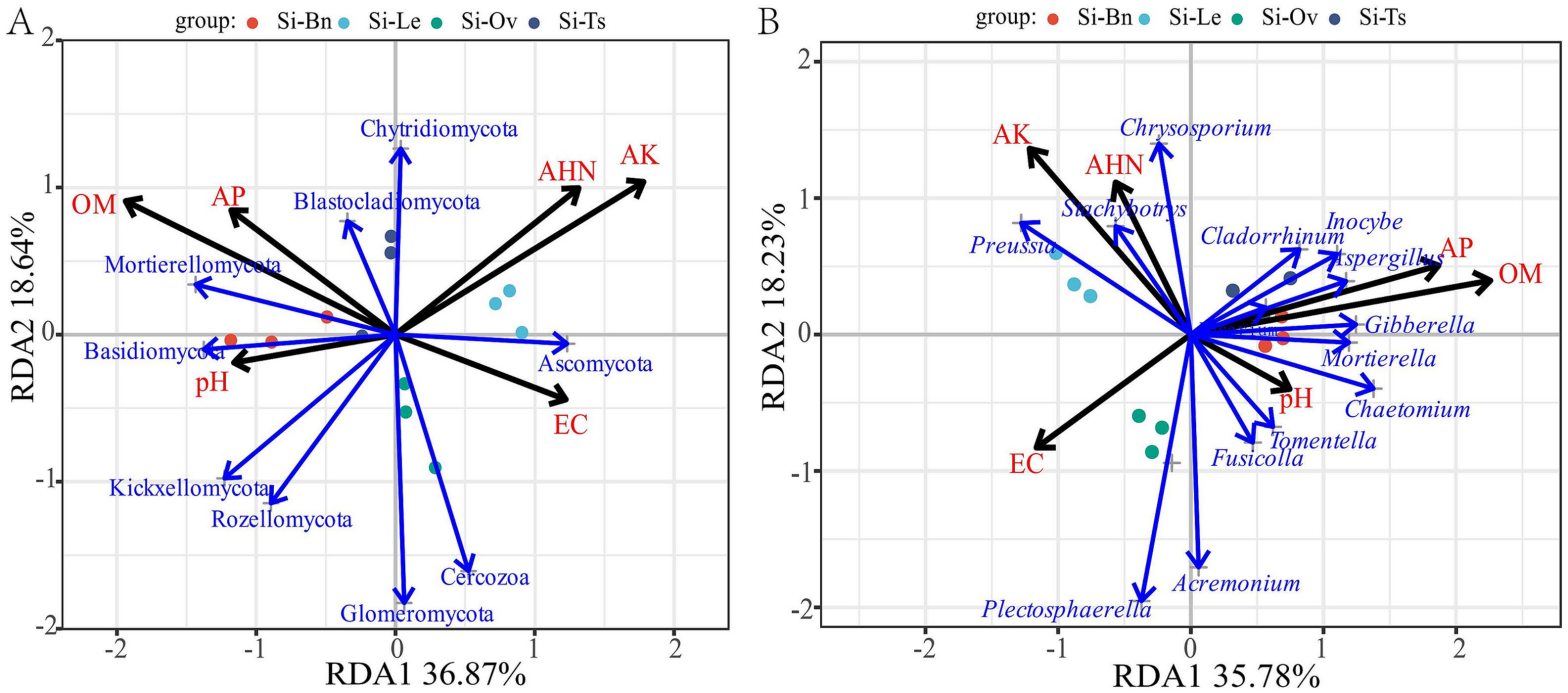
Redundancy analysis (RDA) examining the relationships between soil properties and fungal-community diversity at the phylum **(A)** and genus **(B)** levels under crop-rotation systems.

Redundancy analysis (RDA) revealed clear associations between soil chemical properties and fungal community composition across the different crop rotation systems. In phyla level, the RDA ordination accounted for 55.51% of the total variance in the fungal community structure, with the first and second axes explaining 36.87 and 18.64%, respectively. Among the measured environmental variables, OM (*r*^2^ = 0.8514, *p* = 0.003) and AK (*r*^2^ = 0.8037, *p* = 0.004) were the most influential environmental drivers orchestrating the shifts in fungal community composition across different rotation systems. Additionally, AHN (*r*^2^ = 0.5663, *p* = 0.028) significantly contributed to the community variation, whereas soil pH, EC, and AP showed no significant individual effects on the overall community assemblage (Figure 4A). In genus level, the RDA ordination accounted for 53.01% of the total variance in the fungal community structure, with the first and second axes explaining 35.78 and 18.23%, respectively. Among the measured environmental variables, OM (*r*^2^ = 0.9158, *p* = 0.001), AP (*r*^2^ = 0.6767, *p* = 0.007) and AK (*r*^2^ = 0.8777, *p* = 0.002) were the most influential environmental drivers orchestrating the shifts in fungal community composition across different rotation systems. Additionally, whereas soil AHN, pH, EC, and AP showed no significant individual effects on the overall community assemblage (Figure 4B).

**Figure 5:**
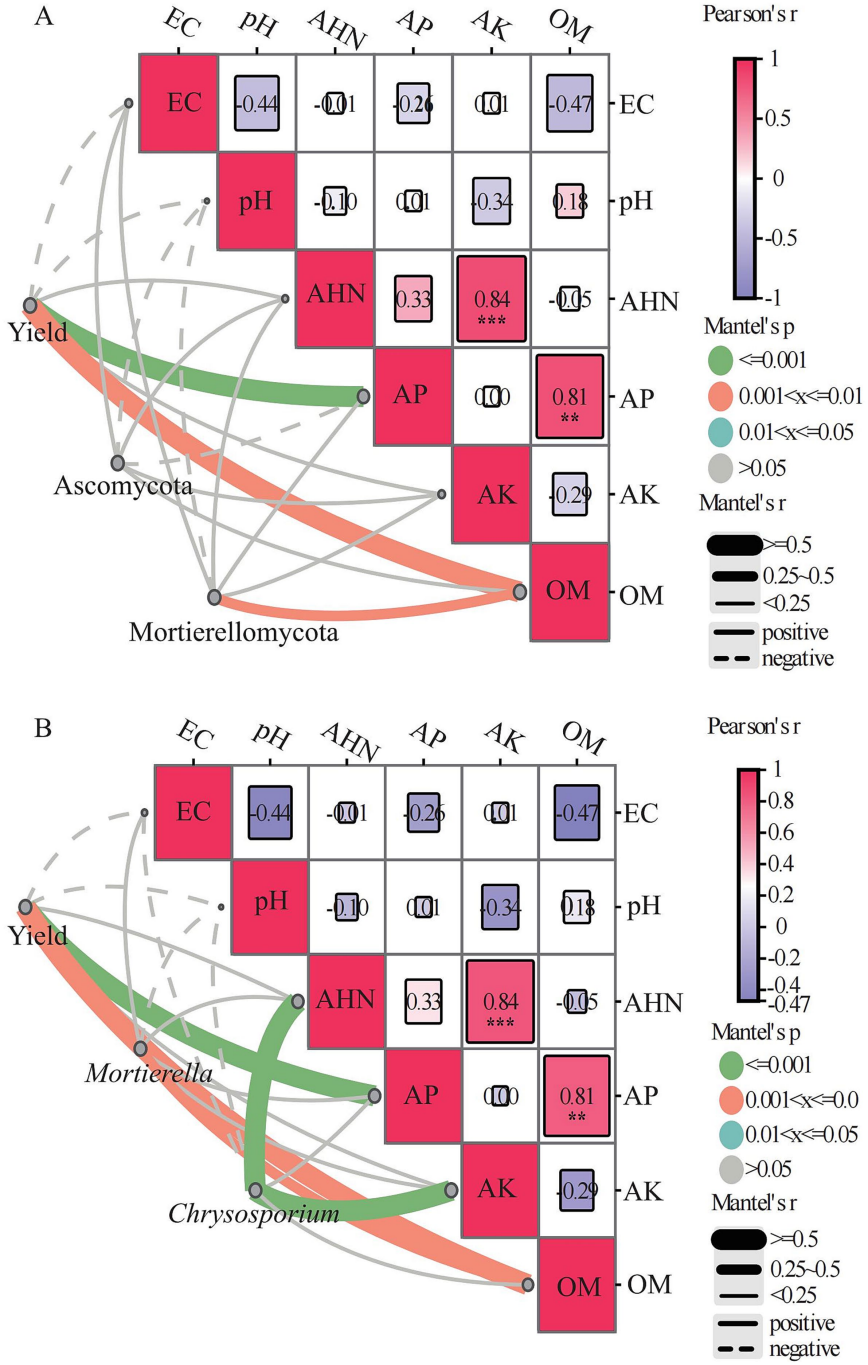
Mantel test analysis examining the relationships among crop yield, dominant fungal phyla **(A)** and genera **(B)**, and soil properties.

The Mantel tests further refined our understanding of the specific linkages between soil properties and biological parameters. Despite its non-significant impact on the soil fungal community structure in the RDA, AP emerged as the most potent predictor of millet yield (*r* = 0.774, *p* = 0.001), followed by OM (*r* = 0.526, *p* = 0.002). This suggests a decoupled response where yield is primarily constrained by phosphorus availability and organic carbon, while the overall fungal community structure is more sensitive to potassium and nitrogen fluctuations. Regarding specific taxonomic groups, the Mortierellomycota phylum exhibited a robust and significant correlation with soil OM (*r* = 0.477, *p* = 0.003), corroborating the RDA results where the Mortierellomycota vector was closely aligned with the OM gradient. In contrast, Ascomycota—the dominant phylum—did not show significant associations with any measured soil properties (*p* > 0.05), suggesting that its assembly may be governed by unmeasured factors or stochastic processes. Furthermore, pearson correlation analysis revealed strong synergistic interactions between several soil nutrients, notably AHN and AK (*r* = 0.84, *p* < 0.001) and OM and AP (*r* = 0.81, *p* < 0.001), indicating that the rotation systems improved soil fertility in a highly coordinated manner (Figure 5A). In genus level (Figure 5B), *Chrysosporium* showed significant correlations with AHN (*r* = 0.79, *p* = 0.001) and AK (*r* = 0.79, *p* = 0.001), and *Mortierella* had a significant positive correlation with OM (*r* = 0.41, *p* = 0.004).

## 4 Discussion

### 4.1 Effects of different crop rotation systems on millet yield

Our results demonstrated that green manure–millet rotation systems, particularly the Si-Ts (millet–Triticale) and Si-Bn (millet–rapeseed) regimes, significantly enhanced millet grain yield and its components compared to the traditional winter fallow system (Si-Le). The remarkable yield increase in the Si-Ts system–reaching a maximum of 85.7% in 2022–suggests a strong cumulative benefit of winter cover cropping on subsequent cereal productivity. Triticale functioned as an effective catch crop, contributing to the retention of residual nitrogen during the off-season and its subsequent release through mineralization coinciding with the period of highest nitrogen demand in the subsequent millet crop (Da Silva et al., 2025). Significant increases in thousand-grain and panicle weights under Si–Ts and Si–Ov systems suggest an optimized source–sink balance, likely mediated by improved moisture conservation and balanced nutrient supply during reproductive phases (Xu H. et al., 2023; Xu J. et al., 2023). Furthermore, the substantial increase in yield components in 2022 compared to 2021 highlights that green manure effects on productivity often intensify over time as soil quality stabilizes (Sun et al., 2025). The fallow-millet system suffered the most significant yield reduction in 2021, potentially due to the challenges associated with continuous cropping, such as pest accumulation and alterations in soil microbial communities (Wang et al., 2022).

### 4.2 Effects of different crop rotation systems on soil properties

The transition from winter fallow to green manure rotations led to a pronounced improvement in soil fertility, characterized by increased OM, AHN, AP, and AK. The elevation of soil organic matter (OM) in the Si-Ts and Si-Bn systems is likely attributed to the consistent input of high-quality organic residues and root exudates, which serve as precursors for stable soil carbon sequestration (Lal, 2004). Available phosphorus (AP) levels were significantly higher in the Si–Ts rotation than in other systems during 2021 and 2022. The integration of Triticale appears to drive the mobilization of recalcitrant phosphorus (P), a process mediated by the secretion of organic acids and the stimulation of phosphatase activity within the rhizosphere (Hasanuzzaman et al., 2018). Compared to the millet–winter fallow system, the integration of Triticale and rapeseed into the rotation significantly augmented topsoil organic matter (OM) content. This sequestration effect is potentially driven by the high carbohydrate inputs from these green manures, which stimulate microbial metabolism and subsequently accelerate the transformation of plant-derived residues into stable soil organic fractions (Liang et al., 2023). Moreover, the significant elevation of AHN and AK levels under Si–Ts regimes indicates that such rotations facilitate an integrated nutrient priming response. We argue that the extensive root architecture of Triticale serves as a biological conduit for potassium pumping from deeper horizons; this vertical translocation facilitates nutrient accessibility within the upper soil layers, directly benefiting the shallow-rooted millet crop (Rmheld and Kirkby, 2010).

### 4.3 Effects of different planting patterns on soil fungal community structure

Fungal taxa constitute a pivotal component of soil microbial consortia; their diversity and compositional architecture serve as critical determinants of soil ecological functionality and resilience (Tian et al., 2025). While diversified crop rotations are known to augment microbial richness and modulate community assembly dynamics relative to monoculture, the specific impacts vary significantly with rotation regimes (Li B. et al., 2023; Li W. H. et al., 2023). The Venn analysis (Figure 1A) showed a substantial enrichment of unique fungal OTUs in the rotation systems, particularly in the Si–Ts (513) and Si–Bn (472) regimes. This pronounced diversification suggests that the incorporation of Triticale and rapeseed broadens the availability of carbon sources and root-derived exudates, which in turn selected unique fungal assemblages and contributed to the pronounced community diversification observed in these regimes (Wang et al., 2025).

The taxonomic composition was overwhelmingly dominated by Ascomycota and Mortierellomycota (Figure 2), while Ascomycota often characterized as versatile saprotrophs, consistently exhibited the highest relative abundance, their dominance significantly decreased in the Si–Bn system compared to the winter fallow control. This decline was mirrored by a robust expansion of Mortierellomycota, which rose from 19.1% under Si-Le to 34.3% under Si-Bn. The proliferation of *Mortierellomycota* is of particular ecological significance, especially within the genus *Mortierella*, are frequently identified as r-strategists that rapidly colonize soils enriched with labile organic matter and are known for their ability to solubilize inorganic phosphorus (Marín-Guirao et al., 2023). Consequently, the observed shifts in the Ascomycota–Mortierellomycota ratio suggest a fundamental transition from a relatively stable, fallow-associated fungal community to a more metabolically active and functionally diverse consortium driven by green manure-derived carbon inputs (Barnes et al., 2016). This microbial reorganization likely underpins the enhanced nutrient priming and superior millet yields observed in these rotation systems.

### 4.4 Relationships between soil properties, fungal community structure, and millet yield

RDA and Mantel test provide a comprehensive mechanistic framework for understanding how crop rotation-induced changes in soil fertility shape fungal community assembly and consequently crop productivity. Although RDA identified soil OM and AK as the primary drivers shaping fungal community composition, the Mantel test revealed that millet yield was predominantly governed by AP and OM availability. This divergence suggests that while the soil fungal consortium is highly sensitive to potassium and carbon fluctuations, the millet yield is constrained specifically by phosphorus-mediated physiological pathways and organic carbon-related soil health.

Soil organic matter (OM) acts as key regulator of the system. The robust OM–Mortierellomycota association (Mantel confirmed; *r* = 0.76) highlights this phylum’s metabolic reliance on labile carbon. As fast-growing saprotrophs, Mortierellomycota thrive in nutrient-rich niches (Li B. et al., 2023; Li W. H. et al., 2023); thus, OM enrichment under Si–Ts and Si–Bn systems likely promoted their reproduction. Furthermore, the OM–AP synergy (*r* = 0.81, *p* < 0.001) suggests a priming effect: organic accumulation stimulates P-solubilizing fungi, directly contributing to increase of AP surge in the Si–Ts system. While available potassium (AK) dominated the RDA, it negatively correlated with minority phyla (e.g., Glomeromycota, Rozellomycota). This implied that high AK restricted the niche space of these taxa while potentially favoring K-tolerant groups within Ascomycota. In contrast, Ascomycota (the dominant phylum) showed positive links only with AHN (*r* = 0.58). Their stability across nutrient gradients maintain dominance via diverse strategies ranging from saprotrophy to endophytism (Oono et al., 2020).

Ultimately, Mantel tests (*r* = 0.774) identify phosphorus availability as the critical factor for yield. Green manure rotations (particularly Si–Ts) efficiently decouple these constraints by simultaneously augmenting microbial diversity (driven by OM/AK/AHN) and crop yield (driven by AP/OM). This demonstrates a synergistic management strategy that not only enriches the fungal community but also secures stable, high-yielding millet production.

## 5 Conclusion

The integration of Triticale, rapeseed, and February orchid in crop rotation with millet has improved topsoil properties and soil fungal communities. This study identified soil OM and AK as the primary drivers shaping fungal community composition and AP and OM availability governed the millet yield. Straw-returning of Triticale to field efficiently increased soil organic matter, which not only boosted the prevalence of beneficial soil fungal communities but also maintained high-yielding millet production. Specifically, the Triticale-millet rotation system has been found to nurture a more diverse and abundant community of beneficial fungi, which is instrumental in sustaining stable and high millet yields. These attributes render the Triticale-millet rotation an optimal model for green manure integration with millet, promoting efficient and sustainable agricultural practices in the lowland plains of Hebei Province.

## Data Availability

The datasets presented in this study can be found in online repositories. The names of the repository/repositories and accession number(s) can be found in the article/Supplementary material.

## Footnotes

1
https://www.chiplot.online/
