# Supplementary material for: Straw-returning of Triticale to field improved the yield of foxtail millet efficiently by modulating soil physicochemical traits and fungal microbiome assembly
Source: Front Microbiol. 2026 Mar 11;17:1791055. doi: 10.3389/fmicb.2026.1791055 (PMC13013490; doi:10.3389/fmicb.2026.1791055)
Supplement: Supplementary file 1 [file Table_1.docx]

Table S1 The components of millet yield under different crop rotation systems

| **Year** | **Crop rotation systems** | **Ear diameter**  **（cm）** | **Panicle weight**  **（g）** | **Grain weight**  **per spike （g）** | **The output rate**  **of the valley（%）** | **Thousand seed weight（g）** |
| --- | --- | --- | --- | --- | --- | --- |
| 2021 | Si-Ts | 2. 1±0. 13a | 20. 31±1. 51ab | 15. 64±0. 71ab | 77. 01±1. 00c | 3. 34±0. 05a |
|  | Si-Bn | 2. 0±0. 15b | 18. 72±0. 30b | 15. 01±1. 00b | 80. 22±1. 08b | 3. 20±0. 10ab |
|  | Si-Ov | 2. 0±0. 20b | 21. 64±1. 35a | 17. 68±1. 04a | 81. 69±1. 53a | 3. 24±0. 05ab |
|  | Si-Le | 2. 0±0. 11b | 19. 03±1. 72b | 14. 69±1. 13b | 77. 20±1. 64c | 3. 02±0. 23b |
| 2022 | Si-Ts | 2. 0±0. 13a | 15. 92±1. 21b | 15. 60±0. 53a | 84. 65±0. 90a | 3. 03±0. 24a |
|  | Si-Bn | 2. 1±0. 10a | 13. 48±1. 35c | 11. 11±0. 85c | 82. 47±1. 67a | 2. 88±0. 09ab |
|  | Si-Ov | 2. 1±0. 08a | 18. 48±0. 72a | 13. 48±0. 50b | 84. 37±1. 30a | 2. 87±0. 18ab |
|  | Si-Le | 2. 0±0. 10a | 13. 60±0. 36c | 11. 19±0. 66c | 82. 42±1. 43a | 2. 65±0. 06b |

Notes: Different letters are significantly different at P < 0. 05 Different lowercase letters indicate significantly differences at the P< 0.05, Si-Bn, millet–rapeseed; Si-Le, millet–fallow; Si-Ov, millet–February orchid; Si-Ts, millet–Triticale.

Table S2 The proportion of enriched differential OTUs in every group

| ID | Si-Le | Si-Bn | Si-Ov | Si-Ts | Phylum | Order | Family | Genus |
| --- | --- | --- | --- | --- | --- | --- | --- | --- |
| OTU_1 | 0.163567 | 0.302841 | 0.195051 | 0.218645 | Mortierellomycota | Mortierellales | Mortierellaceae | *Mortierella* |
| OTU_1102 | 0.007533 | 0.011303 | 0.010474 | 0.014642 | Ascomycota | Hypocreales | Nectriaceae | *Gibberella* |
| OTU_118 | 0.000295 | 0.004504 | 0.000637 | 0.003859 | Basidiomycota | Agaricales | Inocybaceae | *Inocybe* |
| OTU_1262 | 0.003297 | 0.001810 | 0.002392 | 0.001679 | Ascomycota | Xylariales | Microdochiaceae | *Microdochium* |
| OTU_1065 | 0.000356 | 0.000404 | 0.000110 | 0.000871 | Ascomycota | Trichosphaeriales | Trichosphaeriaceae | *Nigrospora* |
| OTU_171 | 0.000075 | 0.000000 | 0.000151 | 0.000795 | Basidiomycota | Agaricales | Crepidotaceae | *Neopaxillus* |
| OTU_1030 | 0.000117 | 0.000295 | 0.000315 | 0.000610 | Ascomycota | Hypocreales | Bionectriaceae | *Bionectria* |
| OTU_1374 | 0.000041 | 0.000075 | 0.000007 | 0.000590 | Basidiomycota | Wallemiales | Wallemiales_fam_Incertae_sedis | *Wallemia* |
| OTU_701 | 0.000075 | 0.000082 | 0.000117 | 0.000576 | Basidiomycota | Cantharellales | Clavulinaceae | *Clavulicium* |
| OTU_1014 | 0.000562 | 0.000302 | 0.000233 | 0.000377 | Ascomycota | Sordariales | Lasiosphaeriaceae | *Podospora* |
| OTU_1071 | 0.000158 | 0.000260 | 0.000274 | 0.000254 | Ascomycota | Sordariales | Lasiosphaeriaceae | *Cladorrhinum* |
| OTU_1031 | 0.000075 | 0.000055 | 0.000130 | 0.000137 | Ascomycota | Sordariales | Lasiosphaeriaceae | *Schizothecium* |
| OTU_1012 | 0.000034 | 0.000027 | 0.000130 | 0.000089 | Ascomycota | Eurotiales | Aspergillaceae | *Aspergillus* |
| OTU_1084 | 0.000123 | 0.000206 | 0.000048 | 0.000075 | Ascomycota | Hypocreales | Bionectriaceae | *Clonostachys* |
| OTU_860 | 0.000014 | 0.000007 | 0.000021 | 0.000062 | Ascomycota | Hypocreales | Hypocreales_fam_Incertae_sedis | *Sarocladium* |
| OTU_692 | 0.000007 | 0.000000 | 0.000021 | 0.000027 | Ascomycota | Pleosporales | Pleosporaceae | *Bipolaris* |
| OTU_1377 | 0.000000 | 0.000000 | 0.000000 | 0.000007 | Glomeromycota | Glomerales | Glomeraceae | *Glomus* |
| OTU_1094 | 0.000000 | 0.000000 | 0.000000 | 0.000007 | Ascomycot | Glomerellales | Plectosphaerellaceae | *Sodiomyces* |
| OTU_1001 | 0.000055 | 0.000021 | 0.000014 | 0.000000 | Ascomycota | Sordariales | Chaetomiaceae | *Chaetomium* |
| OTU_273 | 0.000000 | 0.000000 | 0.000007 | 0.000000 | Ascomycota | Onygenales | Onygenales_fam_Incertae_sedis | *Chrysosporium* |
| OTU_120 | 0.000055 | 0.000014 | 0.000000 | 0.000000 | Chytridiomycota | Rhizophydiales | Rhizophydiales_fam_Incertae_sedis | *Coralloidiomyces* |
| OTU_1213 | 0.000007 | 0.000007 | 0.000000 | 0.000000 | Ascomycota | Pleosporales | Sporormiaceae | *Preussia* |
| OTU_580 | 0.000021 | 0.000000 | 0.000000 | 0.000000 | Ascomycota | Eurotiales | Trichocomaceae | *Talaromyces* |
